# Integrating multiple genomic data: sparse representation based biomarker selection for blood pressure

**DOI:** 10.1186/s12919-016-0044-7

**Published:** 2016-10-18

**Authors:** Hongbao Cao, Wei Guo, Haide Qin, Mengyuan Xu, Benjamin Lehrman, Yu Tao, Yin-Yao Shugart

**Affiliations:** Unit on Statistical Genomics, Division of Intramural Research Programs, National Institute of Mental Health, National Institutes of Health, Building 35, Room 3A 1000, 35 Convent Drive, Bethesda, MD 20892 USA

## Abstract

**Background:**

Although many genes have been implicated as hypertension candidates, to date, few studies have integrated different types of genomic data for the purpose of biomarker selection.

**Methods:**

Applying a newly proposed sparse representation based variable selection (SRVS) method to the Genetic Analysis Workshop19 data, we analyzed a combined data set consisting of 11522 gene expressions and 354893 single-nucleotide polymorphisms (SNPs) from 397 subjects (case/control: 151/246), with the aim to identify potential biomarkers for blood pressure using both gene expression measures and SNP data.

**Results:**

Among the top 1000 variables (SNPs/gene expressions = 575/425) selected, the bioinformatics analysis showed that 302 were plausibly associated with blood pressure. In addition, we identified 173 variables that were associated with body weight and 84 associated with left ventricular contractility. Together, 55.9 % of the top 1000 variables showed associations with blood pressure related phenotypes(SNP/gene expression =348/211).

**Conclusions:**

Our results support the feasibility of the SRVS algorithm in integrating multiple data sets of different structure for comprehensive analysis.

## Background

The determinants of blood pressure (BP) are likely to be a complex combination of genetic, environmental, and other potential confounders including age, gender and smoking status [1]. Moreover, it has been documented that heritability accounts for one-third to two-thirds of the variability in BP [2].

Genome-wide association studies (GWAS) [3–6] and gene expression studies [7] have been conducted to identify biomarkers, such as single-nucleotide polymorphisms (SNPs) and gene expression, associated with BP phenotypes. Although many genes have been reported as hypertension candidates [8], to date, a limited number of studies have integrated different types of genomic data to select biomarkers.

Here we used a sparse representation based variable selection (SRVS) method [9] to integrate a gene expression data set and a SNP data set acquired from the same subjects, for the purpose of identifying BP related biomarkers, and facilitate the understanding of genetic mechanism of the BP disease. The SRVS method has been shown to be feasible in identifying schizophrenia candidate biomarkers, while integrating functional magnetic resonance imaging data and SNP data [10]. It has also been demonstrated that the use of multiple data types may provide higher power to identify potential biomarkers that would be missed by using independent data analysis [11].

## Methods

### Data description

The data set was provided by the Genetic Analysis Workshop 19. Phenotypes were measured at 4 time points, including age; hypertension diagnosis (HD; yes = 1; no = 0); Systolic Blood Pressure (SBP); Diastolic Blood Pressure (DBP); medication status (MS); smoking status (SS; yes = 1/no = 0) and gender. The expression data set consisted of 647 subjects with 16383 expression probes. In the SNP data set, there were 959 subjects with 472049 SNPs, measured from the odd numbered chromosomes (1 ~ 21). In the current study, we used the data obtained from the third examination that has roughly balanced hypertension/non-hypertension numbers (Table 1). This data set included 397 subjects from 46 families having both SNP data and gene expression data. For simplicity, we deleted gene expression probes and SNPs with no associated gene, resulting in a combined data set of *X* ∈ *R* ^397×(11522 + 354893)^ (397 subjects with 11522 gene expression probes and 354893 SNPs). Table 1 summarizes the data set and their clinical measures (age, sex, HD, SBP, DBP, MS, SS).

**Table 1:** Descriptive statistics of data set

	Data set
Subject Number (m)	397
SBP (meanSD)	125.218.0
DBP (mean SD)	70.810.3
Hypertension cases	151
Age (mean SD)	47.714.1
Sex (male)	167
MS (taking drug)	113
SS	66

### Sparse representation-based variable selection

We used 2 regression models to describe the relationship between BP and 6 impact factors: Age, Sex, MS, SS, SNP and gene expression variation.

1 $$ BP={\displaystyle {\sum}_{i=1}^4{\delta}_{\mathrm{i}}{X}_i}+y $$

2 $$ y=\left[{X}_5,{X}_6\right]\left[\begin{array}{c}\hfill {\delta}_5\hfill \\ {}\hfill {\delta}_6\hfill \end{array}\right]+\varepsilon =X\delta +\varepsilon $$

Where *X*_*i*_ for *i* = 1~6 are the 6 impact factors; *δ*_*i*_ are the regression coefficients for each factor. In this study, *BP* ∈ *R* ^*m*×1^ is the BP measurement, SBP or DBP, where *m* is the number of subjects; *X*_1_~*X*_4_ ∈ *R* ^*m*×1^ are Age, Sex, MS and SS, respectively; *δ*_1_~*δ*_4_ ∈ *R* ^1×1^; *X*_5_ ∈ *R* ^*m*×11522^ represents the gene expression measures and *δ*_5_ ∈ *R* ^11522×1^; *X*_6_ ∈ *R* ^*m*×354893^ represents the SNPs and *δ*_6_ ∈ *R* ^354893×1^; *ε* ∈ *R* ^*m*×1^ is the residual vector. *X* ∈ *R* ^*m*×*n*^ is the genetic data matrix integrating both gene expression data and SNP data; *n* = 11522 + 35893 represents the total number of gene expression probes and SNPs; columns of *X* are normalized to have unit L2 norm. $$ \delta =\left[\begin{array}{c}\hfill {\delta}_5\hfill \\ {}\hfill {\delta}_6\hfill \end{array}\right]\in {R}^{n\times 1} $$ is the solution to be found.

Here, we used the Linear Least Squares (LLS) method to solve the linear regression given by Eq. (1) and acquire the residual *y*.

In this analysis, we assumed that only a small number of variables (eg, gene expressions or SNPs) were closely associated with the phenotype (BP). Therefore, the underdetermined linear regression problem given by Eq. (2) becomes a sparse problem aiming to find a sparse solution *δ*, with a few non-zero entries corresponding to BP related genetic variables.

Considering *n* ≫ *m*, we employed a SRVS method, proposed by [10] to solve Eq. (2) and identify potential biomarkers (gene expressions/SNPs) associated with BP.

#### Sparse representation-based variable selection algorithm

1. Initialize *δ* ^(0)^ = 0;
2. For Step *l*,randomly shuffle X with Fisher-Yates algorithm [12]; Then separate X into sub-matrixes in size *m* × *k*; denote those sub-matrixes as *X*_*l*_ ∈ *R* ^*m*×*k*^;
3. Solve the following *L*_*p*_ minimization problem to get the optimal sparse solution *δ*_*l*_ ∈*R* ^k×1^ for each sub-matrix *X*_*l*_:
  3 $$ min\left\Vert {\delta}_l\left\Vert {}_p\right. subject\;to\;\left\Vert y-{X}_l{\delta}_l\left\Vert {}_2\right.\le \varepsilon \right.\right.; $$
4. Update *δ* ^(*l*)^ ∈ *R* ^*n*×1^ with *δ*_*l*_: *δ* ^(*l*)^ (*I*_*l*_) = *δ* ^(*l*-1)^ (*I*_*l*_)+*δ*_*l*_; where *δ* ^(*l*)^ (*I*_*l*_) and *δ* ^(*l*-1)^ (*I*_*l*_) denote the *I*_*l*_ th entries in *δ* ^(*l*)^ and *δ* ^(*l*-1)^, respectively;
5. If a stopping rule is not satisfied, update *l = l +* 1 and go to Step 2. Otherwise, set *δ* = *δ* ^(*l*)^/*l* and terminate. The non-zero entries in *δ* correspond to the column vectors selected, that is, variable selection.

In Step 2, the column number of sub-matrixes X_1_ is chosen according to Cao et al. [10]. In Step 5, we set the following 2 stopping rules: a.)‖*δ* ^(*l*)^/*l* − *δ* ^(*l* − 1)^/(*l* − 1)‖_2_ < *α*, where α is a predefined threshold; and b.) The probability that each pair of column vectors in *X* compared should be greater than 1-*p*_s*top*_. The algorithm terminates when both rules are satisfied, which decides the total number of iterations. The Matlab software toolbox for the proposed SRVS algorithm has been made available online: http://hongbaocao.gousinfo.com/Software4Download.html.

### Bioinformatics analysis

For each top gene selected, we used a biomedical data analysis tool, the Rat Genome Database (RGD) for bioinformatics analysis. The bioinformatics analysis was based on Human Genome Assembly GRCh37 (Genome Reference Consortium Human genome build 37) [13]. The input into RGD are the genes selected (the selected SNP/expression corresponded genes). The outputs include the quantitative trait locus (QTL) study name, logarithm of odds (LOD) score, p value trait and sub-trait. Significant variables (SNPs/gene expressions with LOD score > 3) were reported.

## Results

### The impact of Age, Sex, MS and SS on BP

**Fig. 1:**
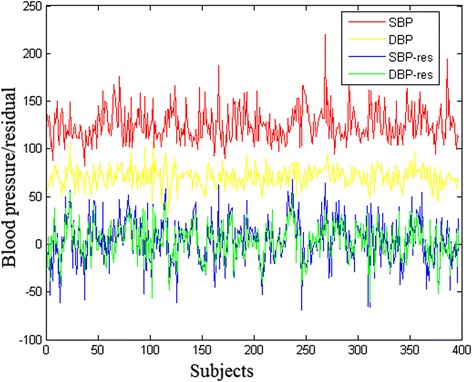
Blood pressure phenotypes of 397 subjects. SBP-res and DBP-res are the residual y of regression problem given by Eq. (1) for SBP and DBP, respectively; x-axis represents the subjects; y-axis represents the blood pressure phenotypes at each subject

Table 2 details the considered regression coefficients: Age, MS, SS and Sex. We obtained these coefficients by solving Eq. (1) using a LLS approach. Figure 1 presents the SBP and DBP measures on the 397 subjects before and after the regression.

**Table 2:** Regression coefficients between BP (SBP/DBP) and 4 clinical measures: Age, MS, SS and Sex

	BP	Age	MS	SS	Sex	Corr before/after regression
m = 397	SBP	1.7	−8.9	13.4	25.5	0.25/0.82
	DBP	0.8	−14.2	10.7	19.7	

The regression coefficients were obtained from linear regression models given by Eq. (3) fitted using the least squares approach. The ‘Corr’ is the Pearson correlation coefficients

It can be seen from Fig. 1 that the residual SBP-res and DBP-res were strongly correlated (Pearson correlation coefficient > 0.82). Therefore, to select BP related genetic variables (SNP/gene expression), we focused on the case using SBP-res as phenotype for Eq. (3).

### Sparse representation-based variable selection

**Fig. 2:**
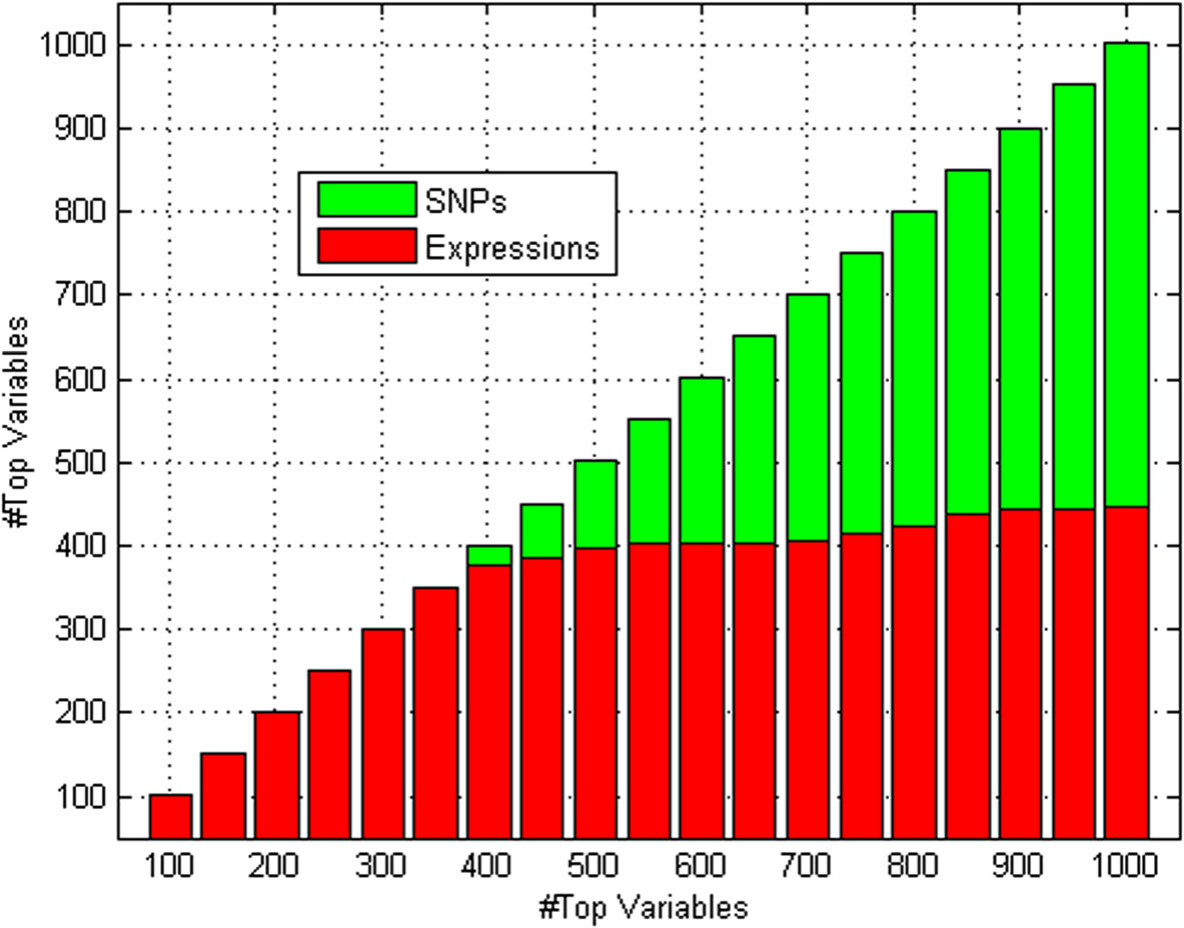
Number of SNPs and gene expressions selected in the top 100 to 1000 variables selected

Figure 2 describes the variable selection results for the data set. Specifically, we analyzed the top 1000 variables (SNPs/gene expressions) selected using the SRVS method from the integrated data set consisting of 11522 gene expression probes and 354893 SNPs. Among those variables, 575 SNPs and 425 expressions were selected, corresponding to 756 genes in total. Figure 2 presents the number of SNPs and gene expressions selected in the top 100 to 1000 variables.

### Bioinformatics analysis

**Fig. 3:**
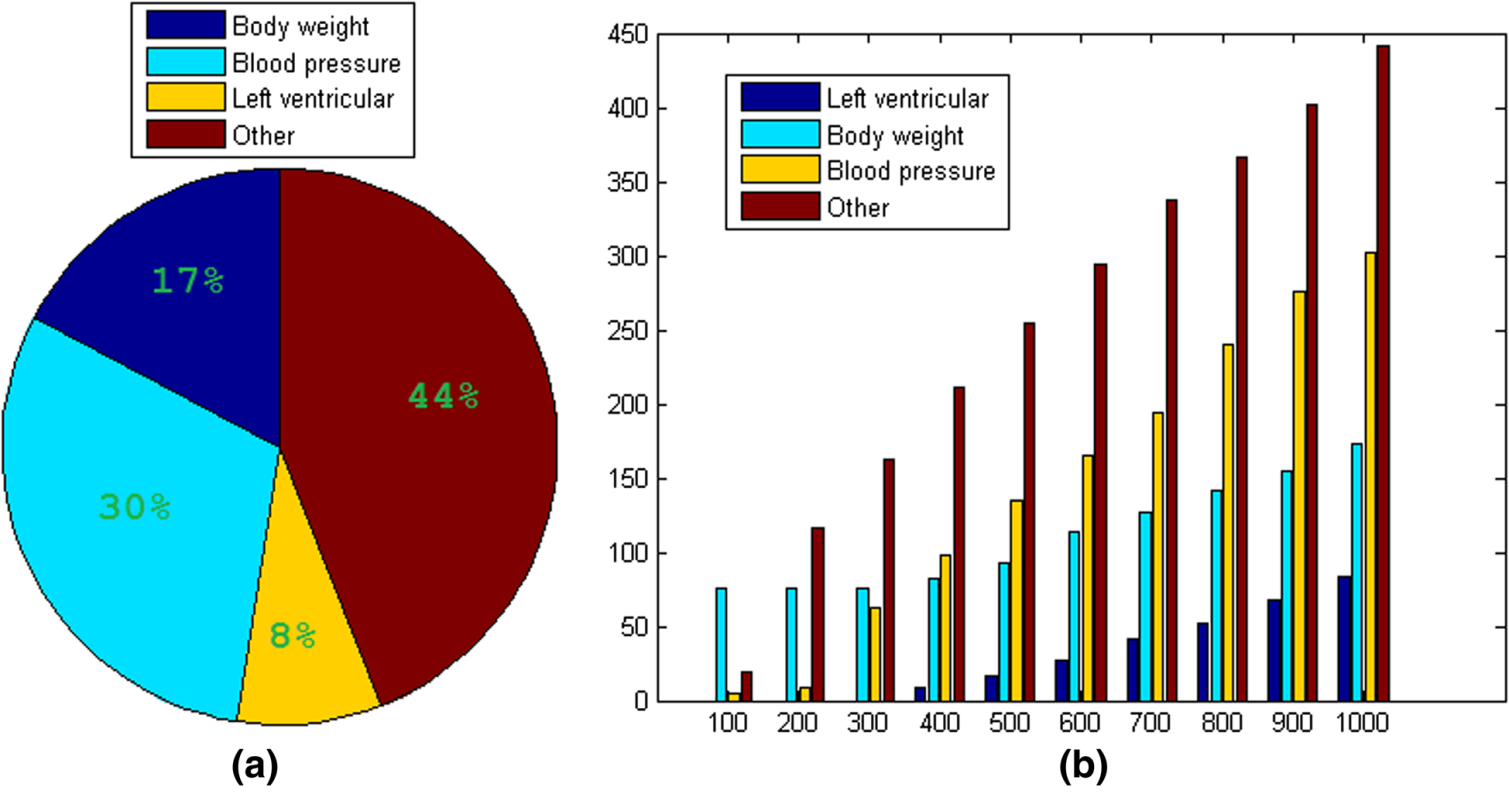
LOD analysis results for the top 1000 variables selected. **a** Pie plot of the variable distribution for the top 1000 variables. **b** Bar plot for the number of variables linked to left ventricular, body weight and blood pressure in the top 100 to 1000 variables selected

For each of the top 1000 variables (SNPs/gene expressions = 575/425), we performed a bioinformatics analysis using RGD as a validation effort, aiming to explore the biological relevance of the selected SNPs and expression signals. Here we define “significant association between genes and disease” as LOD score greater than 3. Figure 3 presents the detailed analysis results. Among those 1000 variables, 302 were plausibly linked to BP (LOD score > 3), 173 were linked to body weight and 84 were associated with left ventricular contractility. Together, 55.9 % of the top 1000 variables revealed association with BP related disease (SNP/gene expression =348/211), corresponding to 330 genes.

## Discussion

In this study, we integrated gene expression and SNP data to select BP related biomarkers using a sparse representation based method—SRVS [10]. The potential influence of 4 covariates on SBP was regressed out and the residuals were then used as the phenotype vector for genomic variable selection. Bioinformatics analysis [13] was performed to study the association of the selected markers/genes to BP-related disease.

Needless to say, in addition to genomic factors, environmental factors also play an important role in BP. Therefore, regressing out their potential influence on BP is necessary for the genomic analysis. In this study, we first calculated the regression coefficients for the regression of SBP and 4confounders: Age, MS, SS and Sex. The results (see Table 2) indicated that BP was positively associated with age, SS and sex, and negatively associated with MS. Nevertheless, age had a weaker impact on BP compared with the other 3 measures, whereas sex seemed to play the most important role among the 4 factors. In addition, the correlations between SBP and DBP before and after regressing out the effect of those influential factors (0.25 vs. 0.82) may indicate that those measures had different influence on SBP and DBP. Because the residual of SBP and DBP after regression showed strong correlation (Pearson correlation coefficient > 0.82; see Fig. 1), we chose to focus on SBP residual based analysis.

Using the SBP residual and integrated data as inputs, the SRVS algorithm ranked the 366415 variables (11,522 gene expression signals and 354,893 SNPs) in descending order, based on their contribution to SBP. We focused on the top 1000 variables. Interestingly, although there are many more SNPs than gene expression probes (354,893 vs. 11,522), a similar number of SNPs and expression signals were selected (SNPs/gene expressions = 575/425). Moreover, the selected gene expression signals dominated the top 400 variables (>90 %), as shown in Fig. 2. This may suggest that gene expression signals are more closely related to the disease phenotype in this data set. However, we would like to point out that non-independence may raise false positive rates in analysis of both SNP data and expression data.

For each of the top 1000 variables selected, we used an online bioinformatics tool RGD to validate the selected variables and identify the biologically meaningful SNPs and expression signals. Among the 425 gene-expression signals selected, approximately half (211/425) of the RGD provided evidence of strong association with BP phenotypes (i.e., body weight, BP and left ventricular contraction), as depicted in Fig. 3. It has been conceptualized that obesity can lead to increased risk of heart disease and high BP [13], while the left ventricle influences the BP directly.

Among the top 500 to 1000 selected variables, more SNPs than gene expression signals were selected, as shown in Fig. 2. In addition, more left ventricular contractility related genes were identified. In total, approximately 60 % of the selected SNPs were identified as “BP related” (348/575) (LOD score > 3). This observation may suggest that, although SNPs are unlikely to directly cause the disease phenotypes, they may affect the development of BP related diseases via regulating RNA expressions.

It should be noted that, while most genes were identified using 1 marker (either SNP or gene expression), some newly identified genes were selected multi-times by different makers. Those genes include GNB1, MEGF6, MMEL1, MORN1, PANK4, PLCH2, PRDM16, PRKCZ, and TP73. These markers are worth further study.

Among the top 1000 variables selected, 44 % do not show strong association with BP (Enrichment LOD score < 3). However, for many of the remaining genes there was evidence of weak linkage (Enrichment LOD > 2) and some demonstrated strong linkage to BP in rat studies [14]. Because of the lack of space, we did not include a detailed discussion of these variables.

Of note, both case and control groups included family members. Although the shared genetic factors may enrich true signals and therefore help to detect potential biomarkers that may be missed in independent subject analysis, this familial correlation may also lead to increased false positives. Therefore, further analysis using independent samples of larger size should be performed to validate the results reported here and to study the correlations between the selected variables. We would like to note that this work focuses more on the feasibility of our sparse algorithm than the discovery of true biomarkers.

## Conclusions

Using our SRVS based integrated analysis of gene expression and SNP data sets, we ranked 11522 gene expression measurements and 354893 SNPs and then performed bioinformatics analysis on each of the top 1000 variables selected. Results showed that 559 variables (SNPs/gene expressions), corresponding to 330 genes, may serve as potential biomarkers for BP related disease (LOD score > 3). Nevertheless, a portion of the selected variables are likely to be false positives. Molecular validation is needed before any solid conclusions can be made. However, results of the current study demonstrate the feasibility of the SRVS algorithm for a comprehensive analysis of multiple data sets of different structure.
